# Supplementary material for: Saccharomyces boulardii (CNCM I-745) improves intestinal damage in sepsis by remodeling intestinal flora structure
Source: Front Cell Infect Microbiol. 2025 Jul 31;15:1602792. doi: 10.3389/fcimb.2025.1602792 (PMC12350329; doi:10.3389/fcimb.2025.1602792)
Supplement: Supplementary file 1 [file DataSheet1.zip › summary/summary/7_Differential_analysis/3_Indicator_result/2_Phylum/Phylum_indicator_Group.pdf]

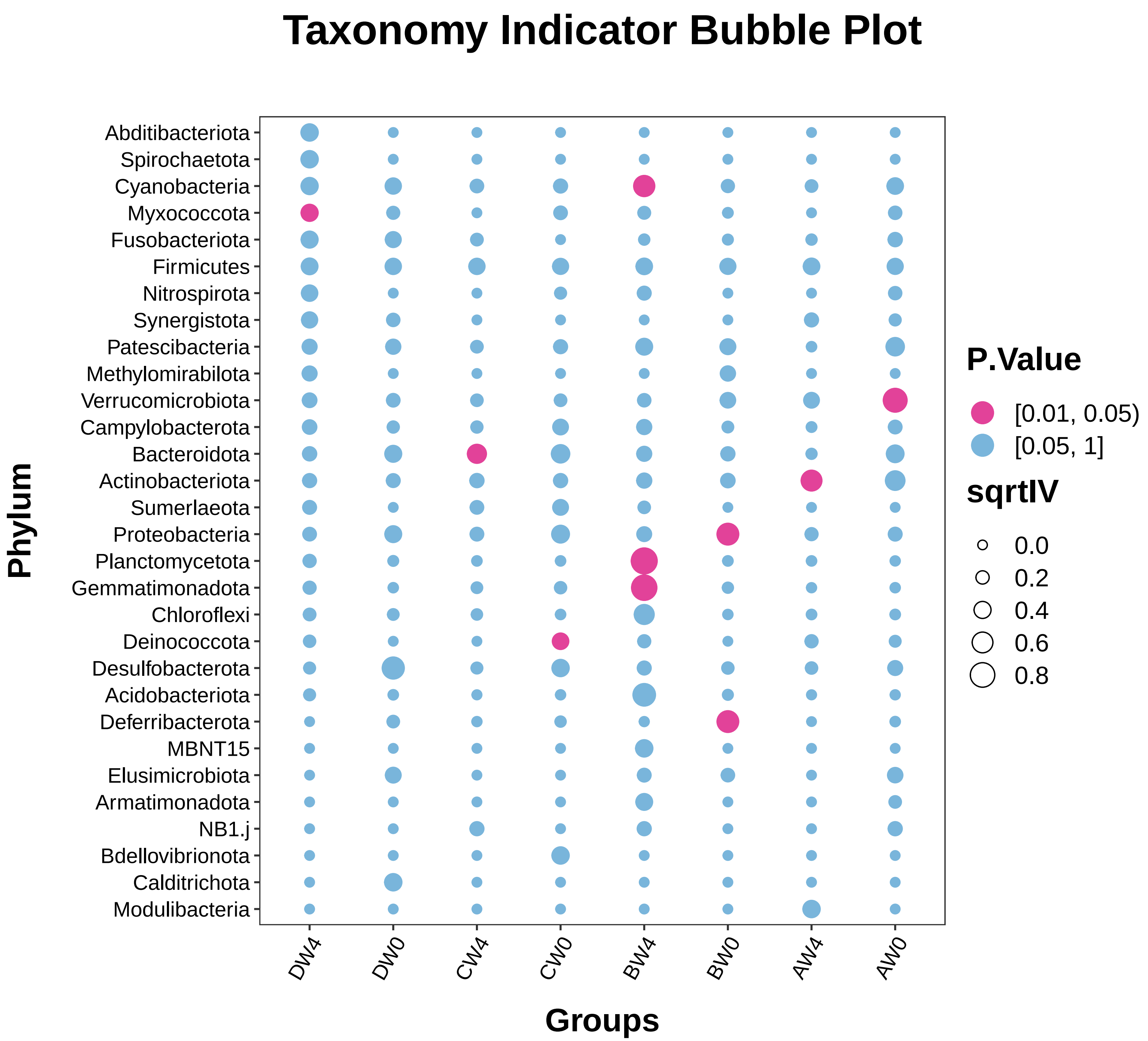
Taxonomy Indicator Bubble Plot
Phylum
Abditibacteriota
Spirochaetota
Cyanobacteria
Myxococcota
Fusobacteriota
Firmicutes
Nitrospirota
Synergistota
Patescibacteria
Methylomirabilota
Verrucomicrobiota
Campylobacterota
Bacteroidota
Actinobacteriota
Sumerlaeota
Proteobacteria
Planctomycetota
Gemmatimonadota
Chloroflexi
Deinococcota
Desulfobacterota
Acidobacteriota
Deferribacterota
MBNT15
Elusimicrobiota
Armatimonadota
NB1.j
Bdellovibrionota
Calditrichota
Modulibacteria
DW4
DW0
CW4
CW0
BW4
BW0
AW4
AW0
Groups
P.Value
[0.01, 0.05)
[0.05, 1]
sqrtIV
0.0
0.2
0.4
0.6
0.8
